# Density of mushroom body synaptic complexes limits intraspecies brain miniaturization in highly polymorphic leaf-cutting ant workers

**DOI:** 10.1098/rspb.2014.0432

**Published:** 2014-06-22

**Authors:** Claudia Groh, Christina Kelber, Kornelia Grübel, Wolfgang Rössler

**Affiliations:** 1 Department of Behavioral Physiology and Sociobiology, Biozentrum, University of Würzburg, Am Hubland, 97074 Würzburg, Germany; 2 Ecological Networks, Technical University of Darmstadt, Schnittspahnstrasse 3, 64287 Darmstadt, Germany

**Keywords:** *Atta vollenweideri*, polymorphism, olfaction, mushroom body, microglomeruli, synaptic plasticity

## Abstract

Hymenoptera possess voluminous mushroom bodies (MBs), brain centres associated with sensory integration, learning and memory. The mushroom body input region (calyx) is organized in distinct synaptic complexes (microglomeruli, MG) that can be quantified to analyse body size-related phenotypic plasticity of synaptic microcircuits in these small brains. Leaf-cutting ant workers (*Atta vollenweideri*) exhibit an enormous size polymorphism, which makes them outstanding to investigate neuronal adaptations underlying division of labour and brain miniaturization. We particularly asked how size-related division of labour in polymorphic workers is reflected in volume and total numbers of MG in olfactory calyx subregions. Whole brains of mini, media and large workers were immunolabelled with anti-synapsin antibodies, and mushroom body volumes as well as densities and absolute numbers of MG were determined by confocal imaging and three-dimensional analyses. The total brain volume and absolute volumes of olfactory mushroom body subdivisions were positively correlated with head widths, but mini workers had significantly larger MB to total brain ratios. Interestingly, the density of olfactory MG was remarkably independent from worker size. Consequently, absolute numbers of olfactory MG still were approximately three times higher in large compared with mini workers. The results show that the maximum packing density of synaptic microcircuits may represent a species-specific limit to brain miniaturization.

## 1. Introduction

Many studies have investigated relationships between the volume of whole brains and/or particular brain centres and the behavioural repertoire, cognitive capacities, social life-style or food preference in a wide range of vertebrate and invertebrate species [1–9]. When relating volumes of brain centres to cognitive capacities, it becomes particularly interesting to quantify brain centres involved in learning and memory, such as the insect mushroom bodies (MBs) [10–15]. In Hymenoptera (a large insect order comprising sawflies, wasps, bees and ants), the MBs can be voluminous in relation to other brain regions [16–22] and contain large numbers of intrinsic neurons (Kenyon cells, KCs) [23–25]. Furthermore, in Hymenoptera, the MBs receive multimodal sensory input (in particular mostly olfaction and vision) segregated into different subdivisions of the MBs [26,27]. Olfactory and visual projection neurons form distinct synaptic complexes (microglomeruli, MG) with KC dendrites in the olfactory lip or visual collar of the MB calyx, respectively [18,22,28–30]. Recent cellular studies of honeybee (*Apis mellifera*) and desert ant (*Cataglyphis fortis*) brains showed that MG undergo structural reorganization associated with brood care, the transition from tasks inside the nest to outside foraging, as well as long-term memory formation [16,18,22,31,32].

Division of labour is fundamental to the efficient functioning of insect societies and is one major determinant of their vast ecological success [33,34]. In the leaf-cutting ant *Atta vollenweideri*, division of labour within the worker caste is associated with worker size and comprises several morphologically distinct worker types specialized in particular tasks (alloethism). Workers exhibit an enormous size polymorphism (up to 200-fold variation in body mass) [35,36]. Mini workers are adapted for tasks inside the dark fungus garden, medium-sized workers forage for food, and large workers have enlarged mandibular muscles specialized for leaf cutting and transport [36]. Although behavioural repertoires may be limited by morphological constraints, a most important basis for behavioural plasticity associated with division of labour resides in the phenotypic plasticity of the nervous system controlling behaviour. For example, recent studies of the primary olfactory centres (antennal lobes, ALs) in *A. vollenweideri* revealed two distinct phenotypes in differently sized workers, in particular, regarding the numbers and volumes of olfactory glomeruli in the ALs [37,38]. The number and/or volume of neurons in primary sensory centres very probably affect processing capabilities in higher order integration centres [39].

We analysed brains of highly polymorphic workers of *A. vollenweideri* at the level of synaptic complexes to address the question of how enormous body size differences associated with alloethism are reflected in the neuronal architecture of brain centres associated with higher order processing. We chose the olfactory subregions of the MBs as olfaction plays a crucial role for leaf-cutting ants. Our general hypothesis is that the volumes of olfactory compartments in the MBs and their microglomerular composition reflect different capacities for learning and memory and/or multimodal integration in mini fungus gardeners compared with larger foragers. As we already have quantitative information on phenotypic plasticity in the primary olfactory centres, the ALs, we chose workers with a head width reflecting the two different AL phenotypes [37]. Pure analyses of absolute and relative brain/neuropil volumes have been criticized [39,40], as it is the absolute number and complexity of neurons and their connectivity that affect neuronal processing capacities [39]. We addressed this general issue of pure volume measurements by using immunolabelling of synaptic proteins and high-resolution confocal imaging to quantify and compare packing densities and absolute numbers of synaptic complexes and relate them to the absolute and relative volumes of the olfactory MB subdivisions.

## 2. Material and methods

### (a) Study animals

We used *A. vollenweideri* (Forel) workers from a laboratory colony collected in 2005 in El Bagual, Formosa, Argentina (by F. Roces). The colony was reared at the Biozentrum, University of Würzburg at 25°C and 40–50% relative humidity in a 12 h day–night cycle, and fed mainly with fresh leaves of privet and dog rose. For the experiments, workers were collected from the fungus chamber and the feeding site.

### (b) Neuroanatomical techniques and immunocytochemistry

Prior to neuroanatomical analyses, the head width (distance between the outer edges of the compound eyes) of workers was used as a measure for individual body size, and determined using a calibrated scale (Wild, Gais, Switzerland) and a stereomicroscope at 25× magnification (Wild M3Z). According to their head widths (*H*_W_), polymorphic workers were classified as mini (*H*_W_ = 0.59–0.71 mm), media (*H*_W_ = 1.01–1.48 mm) and large workers (*H*_W_ = 2.50–3.35 mm).

#### (i) Neuronal tract tracing

A fluorescent dye was injected in the antennal or optic lobes of large *A. vollenweideri* workers to determine the projection areas of olfactory and visual projection neurons in the MB calyces. Ants were anaesthetized with CO_2_, and the heads and antennae were fixed with dental wax (Surgident, Sigma Dental Systems, Handewitt, Germany) in customized acrylic blocks. The heads were entirely covered with cold ant saline solution (127 mM NaCl, 7 mM KCl, 1.5 mM CaCl_2_, 0.8 mM Na_2_HPO_4_, 0.4 mM KH_2_PO_4_, 4.8 mM TES, 3.2 mM Trehalose, pH 7.0). The head capsule was opened by cutting a window between the compound eyes, and glands and tracheae were removed. The broken tip of a pulled glass electrode was coated with small dye crystals of rhodamine dextran with biotin (3000 MW, lysine fixable; Microruby, D-7162, Molecular Probes, Eugene, OR, USA) dissolved in distilled water. The dye was applied manually to the optic lobes or ALs by poking the electrode into the respective neuropils. After dye application, the brains were rinsed with fresh ant saline solution and the heads sealed with parafilm. All animals were kept alive in a moist dark chamber for 2–4 h at room temperature. Brains were dissected, fixed in cold 4% formaldehyde (FA, menthol free, 28908, Fischer Scientific, Schwerte, Germany) in 0.01 M phosphate-buffered saline (PBS, pH 7.2) overnight at 4°C and further processed using a protocol for synapsin immunolabelling of whole mount preparations [29].

#### (ii) Visualization of synaptic complexes in brain sections and whole mounts

The animals were anaesthetized with CO_2_ and decapitated. The heads were fixed in dental wax-coated dishes and covered with cold ant saline solution. After dissection, brains were immersed in 4% FA overnight at 4°C, and then washed in PBS (3 × 10 min). Brains were further processed following two methods: (i) to analyse synaptic complexes (MG) in the MB calyx, pre- and postsynaptic sites of individual MG were visualized in agarose embedded brain sections (100 µm thickness) using a double labelling technique including a monoclonal antibody against the *Drosophila* synaptic vesicle associated protein synapsin I (1 : 10, SYNORF1; kindly provided by Dr E. Buchner, University of Würzburg, Germany) and f-actin phalloidin labelling (0.2 units of Alexa Fluor 488 phalloidin, Molecular Probes, 12379, Leiden, The Netherlands; [18,41]). As synapsin is associated with synaptic vesicles in presynaptic terminals, anti-synapsin antibodies label the central boutons of individual MG. Phalloidin binds to f-actin that is most abundant in dendritic spines and labels the postsynaptic site of one MG [18]; and (ii) to analyse the MB volumes and densities of synapsin positive boutons in the MB calyx, brains were processed following a protocol for whole mount synapsin immunolabelling [29].

### (c) Laser scanning confocal microscopy, image processing and data acquisition

Agarose sections (for high-resolution analyses of individual MG) and whole mount preparations (for volume analyses of the MBs and MG numbers) were viewed using a laser scanning confocal microscope (Leica TCS SP2 AOBS, Leica Microsystems AG, Wetzlar, Germany; objective lenses: 10×/0.4 NA imm, 20×/0.7 NA imm, 63×/1.4 NA imm). Optical sections were taken at a resolution of 1024 × 1024 pixels. For high-resolution analyses of individual MG in double-stained agarose sections, single optical sections were taken at a defined plane within a section of the central brain. At this plane, the MB calyces, the MB pedunculi and the upper and lower division of the central body were clearly visible. The innermost branch of the right medial calyx was scanned at a high resolution to visualize MG within a small outer and a large inner region of the lip (63×/1.4 NA imm, digital zoom 4; figure 2*g*–*i*, and see below). For volume scans, whole brains were scanned as series of sections. For neuronal tract tracing and for volume measurements, optical sections were taken at intervals of 3 µm (10×/0.4 NA imm or 20×/0.7 NA imm with adjusted digital zoom). To quantify synapsin positive boutons within selected volume samples within the lip, the innermost part of the right medial calyx was scanned at high resolution through a depth of 10 µm at intervals of 0.5 µm (63×/1.4 NA imm, digital zoom 2; figure 2*k*).

Image stacks were further processed using three-dimensional software (AMIRA v. 5.3; FEI Visualization Sciences Group, Düsseldorf, Germany). For volume measurements, whole brains and selected neuropils were reconstructed with the help of the interpolation function. For the quantification of projection neuron (PN) boutons, synapsin labelled boutons were counted in defined volumes in two different regions of interest using the AMIRA landmark viewer: four volumes per dense lip region in the outermost layer of more densely arranged synapsin positive boutons (51 × 51 voxel, 374.4 µm^3^) and in the central non-dense lip region (101 × 101 voxel, 1468.5 µm^3^), respectively (boxes in figure 2*k*). Only boutons with outlines mostly within those volumes were included in the counts. In both lip regions, bouton numbers were averaged separately for each individual, and then a mean was calculated for each group. To estimate the total bouton number for all lip regions per brain, the mean bouton numbers were extrapolated to the total lip volume and multiplied by four.

All statistical analyses were performed with Statistica v. 10 (Stat-Soft, Tulsa, OK, USA). Graphs and figures were edited using Corel Draw X3 (Corel Corporation Ltd., Ottawa, Canada), and, in some cases, adjusted for brightness and contrast.

## 3. Results

### (a) Olfactory and visual input to the mushroom body calyces

**Figure 1.**
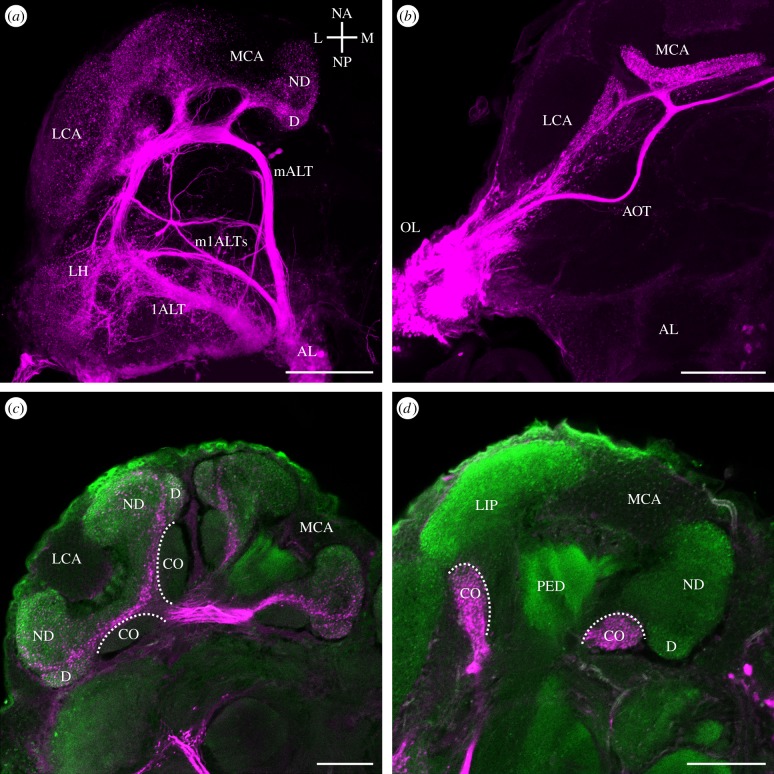
Projection views of anterograde mass fills (rhodamine dextran with biotin, magenta) of AL and optic lobe (OL) output tracts and their projections in higher brain centres. (*a*) The m- and lALT project to the medial and lateral MB calyx (MCA and LCA) and to the LH. Small mlALTs project directly to the LH. Axes (according to [42]): lateral (L), medial (M), neuraxis anterior (NA), neuraxis posterior (NP) apply to the ant's right brain half. (*b*) The AOT bifurcates and extends to both calyces before crossing the midline. (*c*,*d*) Double labelling with an antibody to synapsin (green) shows the relative size of the large olfactory lip (*c*) and the small visual collar (*d*). AOT, anterior optic tract; CO, collar; D, dense lip; lALT, lateral antennal lobe tract; LCA, lateral calyx; LH, lateral horn; mALT, medial antennal lobe tract; MCA, medial calyx; mlALT, mediolateral antennal lobe tract; ND, non-dense lip; PED, peduncle. Scale bars, 100 µm (*a*,*b*) and 50 µm (*c*,*d*).

To be able to clearly distinguish the visual and olfactory subdivisions of the MB calyx, we labelled the antennal lobe tracts (ALTs; tract nomenclature after [42]) of the worker AL using rhodamine dextran injections (*n* = 9). A representative example of projections from the AL through the protocerebral lobe to their target neuropils is shown in figure 1*a*. AL PNs projected via two prominent tracts, the m- and lALT to the MB calyx lip and the lateral horn (LH), and a set of small mlALTs projected directly to the lateral protocerebrum, especially the LH. Axons of these projections were always confined to the ipsilateral half of the brain. Rhodamine dextran fills in the optic lobes (*n* = 8) revealed projections via the anterior optic tract (AOT; tract nomenclature after [42]) resembling the anterior–superior optical tract (asOT) in the honeybee or *Atta sexdens* that contains axons from the medulla and lobula (figure 1*b*; [26,27]). The neurite bundles bifurcated and innervated both ipsilateral MB calyces. In addition, the AOT continued across the midline of the brain (referred to as the anterior commissure [27]). Anterograde labelling in the medulla always revealed projections to the ipsi- and contralateral MB calyces.

**Figure 2.**
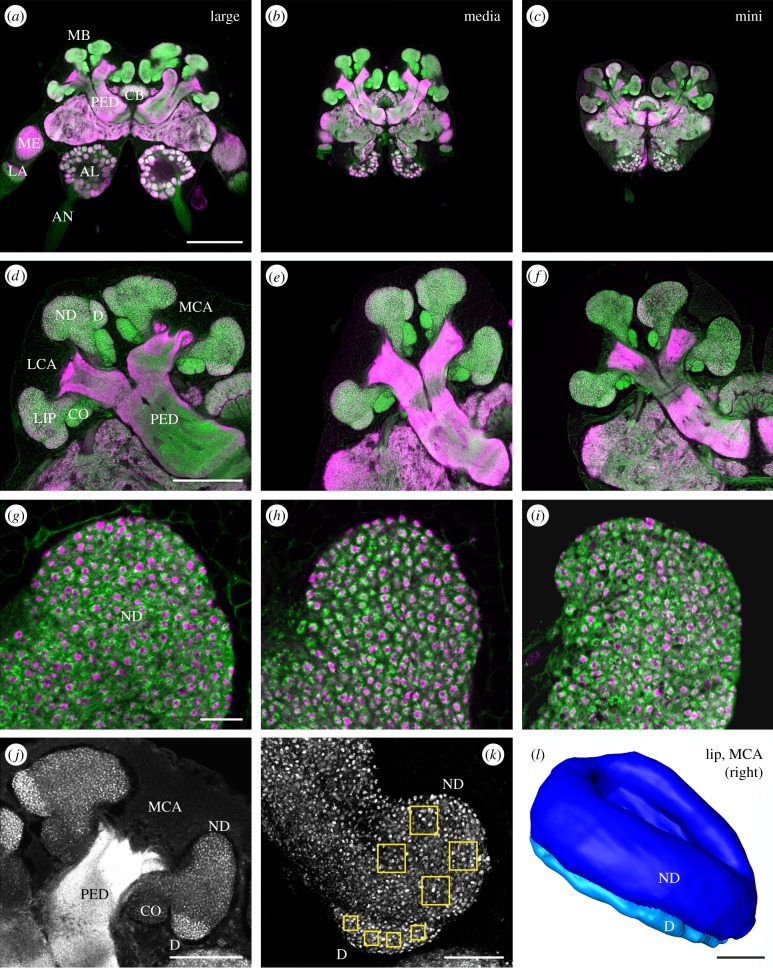
Immunofluorescence labelling and three-dimensional reconstruction of the MB calyx of polymorphic workers. (*a*–*i*) Frontal views of a dorsal/central plane in brains of a large, media and mini worker brain labelled with anti-synapsin (magenta) and fluorophore-conjugated phalloidin (green). (*a*–*f*) Large workers have larger brains and larger MB calyces than media and small workers. (*g*–*i*) Density of calycal MG appears similar in differently sized workers. (*j*,*k*) Synapsin labelled whole mount preparations. (*j*) Volume of the ND and D lip was quantified in the MCA of the right brain hemisphere. (*k*) Enlarged view of the innermost part of the right MCA. Regions of interest used to quantify synapsin labelled boutons in the dense (D) and non-dense (ND) lip are indicated (yellow squares). (*l*) Frontal view of volume reconstruction of the ND (dark blue) and D (light blue) lip rendered from confocal image stacks. AL, antennal lobe; AN, antennal nerve; CB, central body; CO, collar; LA, lamina; LCA, lateral calyx; MB, mushroom body; MCA, medial calyx; ME, medulla; PED, peduncle. Scale bars, 250 µm (*a*–*c*), 100 µm (*d*–*f*), 10 µm (*g*–*i*), 50 µm (*j*,*l*) and 25 µm (*k*).

Many ants are not particularly visual animals [43,44]. In *A. vollenweideri* workers, this was clearly reflected by a small proportion of the MB calyx collar compared with the large lip region as visualized by tract tracing and anti-synapsin double labelling (figure 1*c*,*d*). The MB lip was considerably larger than the collar and was divided into two discrete zones regarding staining intensities: a narrow outer (strongly labelled) and a large inner region (weaker labelled; see also figure 2*d*–*f*, *j*). This pattern suggests that the lip is subdivided into two zones defined by innervations by mALT (outer zone) and lALT projection neurons (inner zone), similar to what has been shown for the honeybee *Apis mellifera* and the carpenter ant *Camponotus floridanus* [45,46]. We defined the two concentric subdivisions of the olfactory lip region as the dense (D) and non-dense (ND) lip. We did not find any consistent layering in tracings of the projections from the optic lobes within the MB calyx collar, similar to results obtained in *A. sexdens* [26].

### (b) Comparison of mushroom body calyx microglomeruli

We analysed brains of mini (*n* = 10), media (*n* = 9) and large (*n* = 10) workers using combined anti-synapsin and f-actin phalloidin labelling in agarose sectioned brains. Confirming previous studies [17], f-actin phalloidin labelled all major central brain neuropils and was largely co-distributed with anti-synapsin labelling (figure 2*a*–*i*). Already at the qualitative level, the comparison of representative frontal views of the brains of a mini, media and large worker at the same scale revealed clear differences in overall brain dimensions (figure 2*a*–*c*). Furthermore, already at this level differences in absolute and relative MB calyx size become apparent suggesting that head size positively correlates with the size of the entire brain, but to a smaller extent with the size of the MB calyces (figure 2*a*–*f*).

In addition to general brain dimensions, the main purpose of double labelling was to estimate possible differences in the density, size and general pattern of synaptic complexes in the MB calyces of differently sized workers. The representative examples demonstrate that in all cases the MB calyces were microglomerular in structure (figure 2*g*–*i*). Each MG comprised a synapsin positive central core surrounded by f-actin phalloidin fluorescence [18,41]. MG within the lip region were arranged in two distinct subdivisions: a smaller outer region with densely packed MG, the D lip (figure 2*d*–*f*; see also synapsin staining in *j*,*k*), and a large region with less densely distributed MG, the ND lip (figure 2*d*–*i*; see also synapsin staining in *j*,*k*). Qualitative evaluation at this level suggests that the MG densities were similar in differently sized workers. To quantify potential differences in the diameter of MG, we determined the mean outer diameter of the phalloidinergic profiles in double labelled agarose sections. The mean diameters of MG did not differ between workers of different size in both the D lip (mini: 2.42±0.4; media: 2.43±0.3 µm; large: 2.35±0.2 µm) and the ND lip (mini: 2.38±0.3 µm; media: 2.38±0.2 µm; large: 2.34±0.2 µm) (data from 20 randomly selected MG from three specimen per group).

### (c) Allometry between the volume of lip subdivisions and the number of synapsin labelled boutons

**Figure 3.**
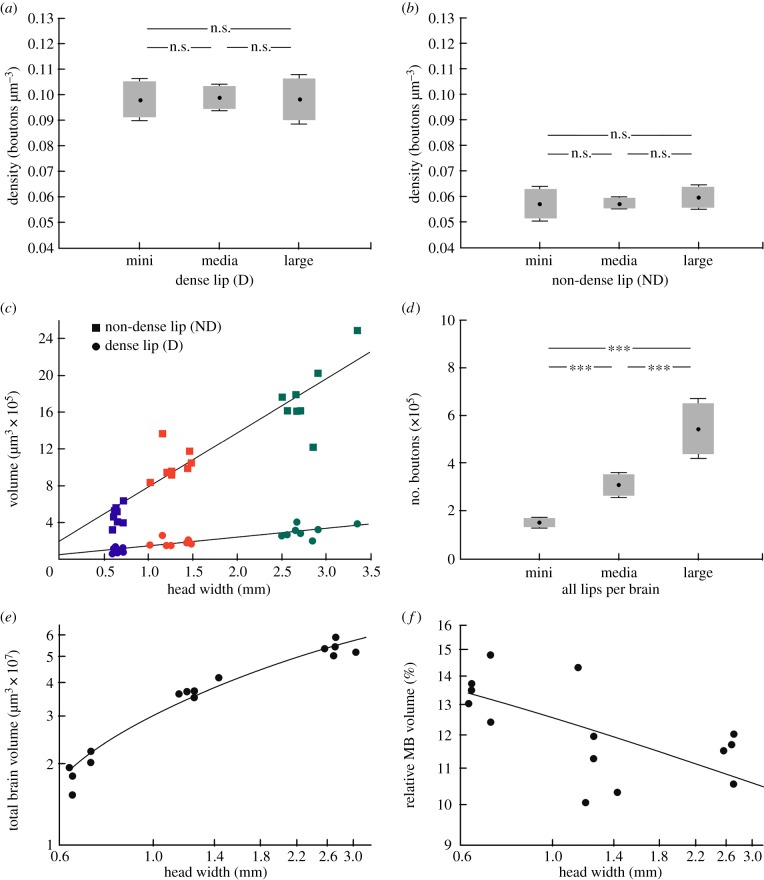
Brain volumes and quantification of synaptic bouton numbers and their packing density based on synapsin labelled whole mount preparations. (*a*,*b*) Projection neuron bouton density is not significantly different in small, media and large workers within (*a*) the D (student's *t*-test; mini–media: *p* = 0.81; media–large: *p* = 0.84; mini–large: *p* = 0.99) and (*b*) the ND lip (student's *t*-test; mini–media: *p* = 0.93; media–large: *p* = 0.22; mini–large: *p* = 0.39). (*c*) Correlation between the head width (*H*_W_) and the volume of the D (linear regression: *y* = 5.88 × 10^5^*x* + 1.76 × 10^5^; *p* < 0.001; *r*^2^ = 0.87; *n* = 24) and ND lip of all calyces (linear regression: *y* = 92181.11*x* + 60408.45; *p* < 0.001; *r*^2^ = 0.80; *n* = 24). (*d*) Number of synapsin labelled bouton numbers extrapolated to all lips per brain is larger in large workers (student's *t*-test; mini–media: *p* < 0.001; media–large: *p* < 0.001; mini–large: *p* < 0.001). (*e*,*f*) Correlation between *H*_W_ and (*e*) the total brain volume (logarithmic regression/Pearson product moment: *y* = 2 × 10^0.7^ ln (*x*) + 3 × 10^0.7^; *p* < 0.001; *r*^2^ = 0.89; *n* = 15) and (*f*) the relative MB volume (linear regression/Pearson product moment: *y* = −0.624; *p* < 0.05; *r*^2^ = 0.39; *n* = 15).

Synapsin immunolabelling in whole mount preparations allowed us to combine quantification of synapsin positive presynaptic boutons in the calyx lip (as a reasonable estimate of MG numbers; [29]) with volume measurements (figure 2*j*–*l*). All raw data for volume measurements and the quatification of synaptic densities are provided in the electronic supplementary material (table S1). To quantify the density of boutons in the D and ND MB-calyx lip, we counted synapsin labelled boutons in defined tissue blocks (four defined volumes of 374.4 µm^3^ per D lip, and four defined volumes of 1468.5 µm^3^ per ND lip; boxes in figure 2*k*) in the inner region of the right medial calyx of mini, media and large workers (*n* = 8 per group). Synapsin labelled boutons in the MB calyx were more densely packed in the D (figure 3*a*) than in the ND lip (figure 3*b*). This difference was found in mini, media and large workers. Most importantly, the density of synapsin labelled boutons in the D lip did not significantly differ between mini, media and large workers (student's *t*-test; mini–media: *p* = 0.81; media–large: *p* = 0.84; mini–large: *p* = 0.99). Similarly, we found no significant difference in the bouton packing density in the ND lip between the three groups (student's *t*-test; mini–media: *p* = 0.93; media–large: *p* = 0.22; mini–large: *p* = 0.39).

To quantify absolute volumes, both lip subdivisions (ND and D) of all MB calyces were measured (figure 2*j*,*l*). The volumes of all ND lips increased significantly with head width (figure 3*c*; linear regression: *y* = 92181.11*x* + 60408.45; *p* < 0.001; *r*^2^ = 0.80; *n* = 24). Compared with mini workers, the mean ND lip volume was twice as large in media workers and more than four times larger in large workers. In the smaller D lip, we also found a statistically significant correlation between the volume of all D lips and the head width of workers (figure 3*c*; linear regression: *y* = 5.88 × 10^5^*x* + 1.76 × 10^5^; *p* < 0.001; *r*^2^ = 0.87; *n* = 24).

Next we wanted to quantify whether the positive correlation between the volume of the lip and the head width of mini, media and large workers was accompanied by an increase in the total number of synapsin labelled boutons in the lip. The extrapolated total number of synaptic boutons in the ND lip of one MB calyx was always higher compared with the D lip and averaged 27 269 (ND mini), 59 423 (ND media) and 106 467 (ND large) compared with 11 067 (D mini), 18 365 (D media) and 30 602 (D large). To estimate the total bouton number for all 4 MB calyx lips per brain, the mean bouton numbers per defined tissue blocks were extrapolated to the volume of the respective lip subregions, added and multiplied by four. We found significant differences in the estimated total bouton numbers of all lips per brain between the three groups (figure 3*d*; student's *t*-test; mini–media: *p* < 0.001; media–large: *p* < 0.001; mini–large: *p* < 0.001). The estimated total number of lip boutons per brain in large workers was three times higher than in mini workers and two times higher than in media workers. Finally, we found a distinct correlation between the head width and the total brain volume (figure 3*e*; logarithmic regression/Pearson product moment: *y* = 2 × 10^0.7^ ln (*x*) + 3 × 10^0.7^; *p* < 0.001; *r*^2^ = 0.89; *n* = 15), which differed by a factor of almost 4 (1.6–6.0 µm^3^ × 10^7^). However, despite a positive correlation of the total brain volume with head width, the relative volume of the MBs was significantly smaller in large workers compared with media and mini workers resulting in smaller MB to total brain ratio in larger ant workers (figure 3*f*; linear regression/Pearson product moment: *y* = −0.624; *p* < 0.05; *r*^2^ = 0.39; *n* = 15).

## 4. Discussion

Using highly polymorphic workers of the leaf-cutting ant *A. vollenweideri*, we relate absolute and relative sizes of a higher processing centre (olfactory MB subdivisions) to the absolute numbers of synaptic complexes to gain information about size-related limitations in the neuronal architecture and resulting capacity of parallel neuronal microcircuits. Our key finding is that the maximum packing density of synaptic complexes (MG) does not differ in differently sized ant workers despite large differences in overall brain size and volumes of the MBs. The remarkably invariable species-specific maximum packing density of MG, therefore, sets a limit to brain miniaturization in mini workers, even with a significant increase of the MB to total brain ratio.

### (a) Definition of lip and collar region in the mushroom body calyx

Anterograde tracing of PNs revealed the representation of olfactory and visual sensory input in subdivisions of the MB calyx in *A. vollenweideri* workers. The difference in size of the two major MB input regions reflects the importance of olfaction compared to vision and also correlates with the size of the antennal and optic lobes [47]. The ALs in *A. vollenweideri* contain high numbers (up to 450) of olfactory glomeruli [37] and make up almost 40% of the major neuropil volume compared with the optic lobes (2–9%) [38]. A large and distinct MB calyx lip region can also be found in closely related species, e.g. *A. sexdens* and *A. cephalotes*, as well as in other, olfactory guided ant species like *Camponotus* ants [46,48]. By contrast, honeybees and visually guided ant species like the desert ant *Cataglyphis fortis* show a reverse lip/collar relation [22,49]. Anterograde tracing of olfactory PNs together with anti-synapsin labelling revealed two distinct zones in the lip, the D and the ND lip region. In the honeybee and in the carpenter ant (*C. floridanus*), the two zones were shown to be related to distinct projection domains of PNs from the medial and lateral ALT [45,46]. The tracing and immunolabelling results confirm the prominence of brain areas devoted to processing of olfactory information in *A. vollenweideri* and correlates well with the importance of olfaction for the behaviour of leaf-cutting ants [33,44].

### (b) Density and size of microglomeruli are similar in differently sized workers

Despite large differences in the absolute volumes, the densities of MG in both subregions of the olfactory MB calyx lip were not significantly different in mini, media and large workers. We used synapsin immunolabelling of whole mount brains to quantify the MG density and to extrapolate the total MG numbers. In addition, double labelling of pre- and postsynaptic compartments of MG in sections showed that the size of individual MG was independent of worker size and MB volumes. These results indicate that the maximum density of MB-calyx MG is species-specific. A recent study shows that density and size of MG in the MB calyx are a species-specific character in different Hymenoptera [17]. The species-specific limit for MG density may be caused by the dimensions of neuronal processes, synapses, glial cells and intracellular organelles, in particular, relatively large mitochondria in synaptic boutons [29]. This may be (among others) one important aspect, as neuronal packing density is likely to be limited by metabolic rates and the required space for mitochondria [50]. However, future studies at the subcellular level are needed to reveal possible adaptations at pre- and/or postsynaptic sites of MG. The D and ND lip regions in *A. vollenweideri* very likely reflect the m- and lALT zones found in the honeybee and carpenter ant. Physiological and ultrastructural analyses in the honeybee showed that lALT neurons respond more generalistically and have a higher synaptic divergence in contrast to a higher odour specificity and less diveregent synaptic circuitry in mALT neurons [29,51]. These differences are likely to put different constraints on the maximum packing densities of microcircuits in both olfactory subregions.

MG within the MB calyx were shown to undergo structural plasticity during adult maturation (nurse forager transition) and after the formation of olfactory long-term memory [14,22,31,49]. In *A. vollenweideri*, differently sized workers show differences in their behaviour. Large workers are equipped with powerful mandibles suited to cut fresh plant material and are specialized in finding digestible plant substrate for the fungus and to transporting the leaf fragments back to the nest [35,52,53]. By contrast, mini workers are specialized in the maintenance of brood and fungus inside the nest, although they can also be found outside the nest on the trails [36,54–56]. Furthermore, it was shown that large workers have higher trail fidelity than mini workers [57]. The differences in pheromone trail following behaviour are reflected in the AL structure. Two different AL phenotypes can be found in *A. vollenweideri*: mini fungus gardeners have a significantly smaller number of olfactory glomeruli, whereas large workers have a larger number of glomeruli and a significantly enlarged glomerulus for processing of trail pheromone information [37,38]. Although MG densities are remarkably invariable in polymorphic workers, the increase in the complexity of AL glomeruli between mini and large workers seems to be reflected in the synaptic architecture in the olfactory MB calyces, as large workers have three times more olfactory MGs in the MBs than mini workers.

### (c) Smaller brains have relatively larger mushroom bodies

Mini workers had the smallest absolute MB volumes, but, in relation to their total brain volumes, the largest relative volume of the MBs compared with media and large workers. By contrast, the volume of the optic lobes is much smaller in mini workers [38]. In mini workers, the MB volumes are disproportionally large, most probably to compensate for miniaturization because of the importance of this brain centre for the behavioural tasks of mini workers [58,59]. Several studies have investigated brain allometry and the significance and consequences of brain miniaturization in different Hymenoptera species [59–62]. Specific brain regions should enlarge only when there is a strong functional necessity [63]. However, comparative studies that invariably quantify relative brain/neuropil volumes have been criticized [39,40], as it is primarily the absolute, not the relative number of neurons, their spatial dimensions, synaptic connectivity and the available energy that affect information processing within the nervous system [39]. Our results show that mini workers have the largest relative size of MBs, but comparison of the absolute numbers of individual synaptic modules within the MBs strongly indicates that the computational capacity of the MBs in mini workers very likely is inferior to those of media and large workers. This underlines the limitation of relative volume measurements and emphasizes the importance of high-resolution analyses at the levels of neurons and synapses to be able to estimate computational capacities of brain neuropils [39].

### (d) Compensation in small brains is limited by a species-specific packing density of synaptic complexes

The estimated absolute number of MGs in all four MB calyx lips ranges between less than 200 000 in mini and more than 600 000 in large workers. Similar total numbers of MG in the olfactory subdivisions of the MB calyx were found in the honeybee (up to 700 000) [29]. Other, non-hymenopteran species show less voluminous MBs with much lower numbers of microglomerular structures [17], for example, in *Drosophila melanogaster* (approx. 2000 MG per brain) [64]. We hypothesize that a lower number of parallel MG circuits in the MBs has consequences for the processing capacities and memory storage abilities. Species-specific differences in MG densities indicate that comparison of absolute and/or relative volumes of brain compartments (in particular, as shown here for the MB) across species need to be interpreted very carefully, especially if they do not include any measure of the neuronal packing densities, numbers and/or other attributes of individual neurons. Furthermore, it needs to be taken into account that additional adaptations or compensations may be present at the ultrastructural and/or molecular levels. If possible, behavioural and/or physiological data need to be added to estimate the computational capacities of particular brain regions. Finally, the body size independent density of synaptic complexes in the MB calyces in a given species does not necessarily imply that this is also the case in other brain regions as the conditions may be different in different brain centres within a species. Potential adaptations and compensations at the level of the neuronal cytoarchitecture and connectivity clearly limit the value of pure volume measurements (absolute as well as relative) as an estimate for the neuronal capacities of brain modules.
